# Controlling Arteriogenesis and Mast Cells Are Central to Bioengineering Solutions for Critical Bone Defect Repair Using Allografts

**DOI:** 10.3390/bioengineering3010006

**Published:** 2016-01-11

**Authors:** Ben Antebi, Longze Zhang, Dmitriy Sheyn, Gadi Pelled, Xinping Zhang, Zulma Gazit, Edward M. Schwarz, Dan Gazit

**Affiliations:** 1 US Army Institute of Surgical Research, Multi-Organ Support Technology, 3698 Chambers Pass, Fort Sam Houston, TX 78234, USA; benantebi@gmail.com; 2 Center for Musculoskeletal Research, University of Rochester, 601 Elmwood Avenue, Rochester, NY 14642, USA; Longze_Zhang@urmc.rochester.edu (L.Z.); Xinping_Zhang@urmc.rochester.edu (X.Z.); Edward_Schwarz@urmc.rochester.edu (E.M.S.); 3 Department of Surgery, Cedars-Sinai Medical Center, Los Angeles, CA 90048, USA; Dmitriy.Sheyn@csmc.edu (D.S.); Gadi.Pelled@cshs.org (G.P.); Zulma.Gazit@csmc.edu (Z.G.); 4 Board of Governors Regenerative Medicine Institute, Cedars-Sinai Medical Center, Los Angeles, CA 90048, USA; 5 Skeletal Biotech Laboratory, Hebrew University-Hadassah Faculty of Dental Medicine, Jerusalem 91120, Israel

**Keywords:** critical bone defect, arteriogenesis, osteogenesis, fibrosis, mast cells, recombinant parathyroid hormone (rPTH; teriparatide; Forteo)

## Abstract

Although most fractures heal, critical defects in bone fail due to aberrant differentiation of mesenchymal stem cells towards fibrosis rather than osteogenesis. While conventional bioengineering solutions to this problem have focused on enhancing angiogenesis, which is required for bone formation, recent studies have shown that fibrotic non-unions are associated with arteriogenesis in the center of the defect and accumulation of mast cells around large blood vessels. Recently, recombinant parathyroid hormone (rPTH; teriparatide; Forteo) therapy have shown to have anti-fibrotic effects on non-unions and critical bone defects due to inhibition of arteriogenesis and mast cell numbers within the healing bone. As this new direction holds great promise towards a solution for significant clinical hurdles in craniofacial reconstruction and limb salvage procedures, this work reviews the current state of the field, and provides insights as to how teriparatide therapy could be used as an adjuvant for healing critical defects in bone. Finally, as teriparatide therapy is contraindicated in the setting of cancer, which constitutes a large subset of these patients, we describe early findings of adjuvant therapies that may present future promise by directly inhibiting arteriogenesis and mast cell accumulation at the defect site.

## 1. Introduction

Overcoming tissue fibrosis and scarring following reconstructive craniofacial and limb salvage surgery remains one of the greatest challenges for patients suffering from birth defects, traumatic injuries, or cancers that invade the musculoskeletal system. While bone tissues have regenerative capabilities that enable self-repair of fractures, in extreme situations that result in a “critical defect” complete regeneration cannot occur. Although several scientific works have defined critical defects based on size alone (e.g., larger than 5 mm for rat calvaria) [1], it is now clear from animal models and early clinical reports that other factors, such as location of the bone defect, adjacent bone quality, soft tissue coverage and vascular supply, are also major determinants [2]. Thus, elucidating the nature of critical defects is essential towards the development of bioengineering solutions to overcome this major clinical problem.

### 1.1. Fracture Repair

Bone tissue retains the unique capacity to regenerate into adult life without scarring through a tightly-regulated physiological process. Fracture repair can be accomplished either directly (*i.e.*, primary) via intramembranous healing, or indirectly (*i.e.*, secondary) via the combination of both intramembranous and endochondral bone repair. Primary healing is ideal since it directly restores the anatomical structure and physiological function of bone (lamellar structure with Haversian systems); however, this rarely takes place spontaneously as it requires anatomical reduction and stable conditions (commonly achieved by a clinician through open reduction and internal fixation) [3]. Most frequently, fracture healing occurs indirectly through a temporal four-step process: (1) hematoma formation; (2) soft callus formation; (3) hard callus formation, and (4) bone remodeling [3,4].

Briefly, following trauma, the damage to bone vasculature leads to the activation of the coagulation cascade to form a hematoma. This hematoma is characterized by an acute inflammatory milieu, consisting of tumor necrosis factor (TNF), interleukin (IL) 1, IL-6, IL-11, and IL-18, as various immune and stem cells are recruited to the injured site. This acute pro-inflammatory response, which peaks at 24 h and lasts approximately seven days, induces angiogenesis, vascular endothelial growth factor (VEGF) production, and the differentiation of recruited progenitor cells to bone-forming osteoblasts and bone-resorbing osteoclasts. The coagulated hematoma also provides a primitive template for the subsequent callus formation to further stabilize the fractured bone. Thus, in the second step of indirect bone healing, a soft callus forms through both intramembranous ossification of the external callus as well as fibro-cartilage formation of the internal callus. This stage is also characterized by angiogenic invasion of capillaries within the callus. In the third step, the soft callus mineralizes to form a hard callus of woven bone while, during the fourth and last step, the hard callus is remodeled into lamellar bone and normal vascular supply is fully restored [5].

### 1.2. Critical Size Bone Defects

A critical size defect is a fracture that is unable to heal or bridge by itself via indirect bone formation, leading to a nonunion. Nonunions are broadly classified into two types: viable and non-viable nonunions. Viable (or hypertrophic) nonunions typically occur due to improper fixation and excessive motion at the fracture, as a result of poor surgical technique or suboptimal hardware. In this type of nonunion, the blood supply, along with the characteristic callus, is present. In contrast, non-viable (or atrophic) nonunions lack sufficient blood supply to the fracture site and as a result are devoid of callus formation [2]. In humans, nonunion is generally defined as the non-consolidation of bone fragments at the fracture site within six months following trauma. Normally, no callus formation or no progress in callus formation after four weeks from initial trauma will potentially result in a nonunion [6]. Importantly, hypoxia is an effective driving force for angiogenesis, and involved in angio-osteogenesis coupling in bone defect healing [7]. Moreover, the degree of hypoxia might differ between defect regions with small vessel-dominant and large vessel dominant ingrowth. As such, strategies or interventions are needed to restore bone in hypoxic settings and in places where bone would otherwise not be present [8].

### 1.3. Autograft and Allograft Interventions

The current standard of care for treating nonunions and high-energy bone traumas is the autograft. Although autograft is ideal for promoting bony unions due to its inherent osteogenic, osteoinductive, and osteoconductive properties, its harvesting is associated with serious clinical complications, such as physical and cosmetic co-site morbidities, chronic postoperative pain, blood loss, and increased risk of surgical site infection. More importantly, the finite amount of autograft that can be harvested from the donor makes its applicability limited [9]. Although allograft is a readily available bone substitute, it lacks the osteogenic and osteoinductive potential of autografts. More importantly, allografts are fraught with limitations, such as disease transmission, increased risk of immunologic reaction, high failure rate (23%–43%), and infection [10]. These shortcomings have inspired investigators to seek other clinical alternatives, such as those developed by tissue engineering approaches [11,12,13]. In this review we will focus on the use of bone allografts as a bioengineering solution for critical bone defect repair. Other reviews can be found that cover alternative approaches that utilize stem cells and biomaterials for a similar goal [14].

## 2. Current Challenges and Approaches for Critical Bone Defect Repair

### 2.1. Growth Factor Delivery

Structural allografts that are used as osteoconductive materials typically fail to bridge large critical defects. Various studies have optimized this approach by coating the allografts with osteogenic and immunomodulating factors. For example, polymer-coated allografts were used to locally deliver an analog of sphingosine-1-phosphate (S1P), a bioactive sphingolipid growth factor that plays physiological roles in immune cell trafficking, vascular network formation and maturation, and osteogenic activities. This S1P-coated allograft was shown to enhance tibia [15,16] and cranial bone defect regeneration [16]. In the same manner, we have explored strategies for revitalizing allografts by local gene transfer of angiogenic, osteogenic and remodeling factors via freeze-dried recombinant adeno-associated virus [17,18,19,20]. Others have attempted to promote allograft integration by coating the necrotic bone with osteogenic proteins [21,22,23]; however, this strategy had questionable success, most probably due to the short half-life and the adverse effects of the high-dosed proteins. Additionally, slow and rapid release of VEGF and BMPs from allografts was attempted to promote vascularization and bone formation [24]. Several studies using live and devitalized femoral isografts (derived from genetically-identical animals) demonstrated significant disparity in cortical neovascularization [17,25]. Whereas robust angiogenesis induces an extensive vascular network throughout the cortical length of live isografts, angiogenesis appears restricted to host-graft junctures in the devitalized isografts [25]. These findings are consistent with the observation that expression of the *VegfA* gene, which encodes VEGF, is delayed and suppressed in implanted processed allografts [17], leading to deficiencies in allograft neovascularization. Dramatic impairment of new bone formation was found when fractured femurs were treated with a soluble VEGF receptor, Flt-IgG, during the course of endochondral ossification and intramembranous bone formation [26].

### 2.2. Intermittent rPTH Administration

Parathyroid hormone (PTH) is an 84 amino acid polypeptide synthesized by the parathyroid glands and acts on kidney and bone to regulate calcium homeostasis via intracellular signals that include cyclic adenosine monophosphate (cAMP), inositol phosphate, and calcium, and activates both protein kinase A and C [27]. Teriparatide is a recombinant form of the 1–34 amino acid portion of human PTH (rPTH). It has a molecular mass of 4117.8 daltons and is manufactured using a genetically modified strain of *Escherichia coli* [28]. In 2002 teriparatide was approved by the FDA for use as an anabolic agent in the treatment of adults with severe osteoporosis at high risk for fracture. Initial human studies with teriparatide have demonstrated its capacity to increase cancellous bone volume and connectivity as well as increase cortical thickness [29,30,31,32,33,34]. Additionally, rPTH was shown to increase endogenous mesenchymal stem cell (MSC) migration to injury sites [35], promote osteoblast progenitor proliferation and differentiation, and decrease osteoblast apoptosis [36,37].

The repair and incorporation of bone grafts commonly used in orthopedic reconstructive surgeries constitute a regulated process that proceeds through several stages. Recently, it was found that treatment with teriparatide could enhance allograft integration [38,39,40,41]. rPTH has been shown to enhance structural allograft healing by: (1) anabolic effects on new bone formation via small-vessel angiogenesis; (2) inhibition of angiopoietin-2-mediated arteriogenesis [42]; and (3) enhancement of osteogenic differentiation via bone morphogenetic protein signaling [36]. The cells undergo distinct stages of differentiation, from pre-osteoblasts to chondrocyte-like osteoblasts that uniquely express chondrogenic and osteoblastic markers and mature to become osteoblasts [43]. It has been found that PTH induces bone formation in structural femoral bone allograft proximity via intramembranous pathway [41] and calvarial bone allografts via membranous regeneration in which mast cells were also found to be affected [44].

Several clinical case reports indicated the healing of fracture non-unions by teriparatide therapy without surgery [45,46,47]. While rPTH-induced new bone formation in these cases, surprisingly it also resolved the fibrous tissue between the fractured bones to achieve healing as quantified by the Union Ratio [46]. A series of follow-up studies show that the critical rPTH treatment period is from 1–3 weeks post allografting [41], which is the transition period from the inflammatory to the anabolic phase of healing. It is also the stage in which new blood vessels produced by angiogenesis remodel into large vessels via arteriogenesis during scarful healing [42]. Studies also showed the remarkable inhibitory effects of rPTH on the host immune, fibrotic and arteriogenic responses during femoral and calvarial allograft healing [42,44]. First, it was noted that rPTH significantly decreased total allograft vascularity compared to placebo. However, upon careful review of the vascular micro-CT and histology data it became clear that rPTH treated allografts contain large numbers of smaller blood vessels, while placebo treated allografts contain fewer vessels that are much larger. Of note is that rPTH inhibition of large vessel arteriogenesis during critical defect healing is consistent with studies by Kang *et al.* [48], which demonstrated that rPTH reverses radiation-induced hypovascularity in the murine mandible undergoing distraction osteogenesis due to small vessel angiogenesis. Interestingly, histology of healing allografts revealed toluidine blue positive mast cells surrounded these large vessels. As these results on both angiogenesis and osteogenesis were phenocopied in transgenic mice that express a constitutive PTH receptor in osteoblastic cells (*Col1*-caPTHR mice) [42], it is clear that rPTH signaling in osteoblasts is responsible for both the anabolic and inhibited arteriogenesis effects for rPTH. Most importantly, and consistent with our “scarless healing” hypothesis, we found that rPTH significantly reduced fibrosis around allografts: from 60% of the total tissue area, down to 31%, which was almost the same as autograft healing (23%) [42]. Additional findings of rPTH effects in the calvaria model were even more remarkable from a scarless healing stand point, as both micro-CT and histology confirmed that allograft healing never results in bony union due to extensive fibrosis, while there was clear evidence of host-allograft bony union in the rPTH treated mice [44]. Importantly, this rPTH treatment also produced the same increase in small vessel angiogenesis, and decreases in large vessel arteriogenesis and mast cell numbers, that was observed in the femur. Thus, rPTH mediated scarless healing of bone occurs in the absence of endochondral ossification, and these findings suggest that the focus should be on the osteoblast at the edge of the defect as the primary target.

## 3. Mast Cells and Critical Defect Repair

### 3.1. Overview of Mast Cells

Mast cells (MCs) are bone marrow-derived leukocytes first described by Paul Ehrlich in 1878 [49]. MCs are granulated cells that circulate the blood in an immature form and later migrate to interstitial tissues to proliferate and differentiate into their mature form [50]. Upon activation, MCs degranulate a composite mixture of histamine, proteases, cytokines, and growth factors to their microenvironment; this makes MCs extremely potent immune cells, a fact exemplified by their notorious role in allergy and anaphylactic shock. MCs play roles in both adaptive and innate immune response and, as such, mainly reside in external body tissues that are prone to infections (*i.e.*, skin, mucosa of the gastrointestinal, respiratory, and genitourinary tracts) as well as in connective tissues [51]. Importantly, MCs also reside in close proximity to vessels, a fact that makes them key players in wound healing, tissue remodeling, fibrosis, and angiogenesis [52]. Indeed, recently, a new stance has emerged—one that attributes MCs a manifold function, including roles in tolerance to graft rejection, autoimmunity modulation, protective immunity against pathogens, cancer progression, metabolic disorders, tissue remodeling, wound healing, and angiogenesis [53,54,55].

### 3.2. Mast Cells in Tissue Repair: Roles in Angiogenesis, Inflammation and Bone Repair

As innate immune cells residing in barrier organs (e.g., skin), MCs play a vital role in host defense by recruiting immune cells, and more specifically neutrophils, to the site of injury [56,57,58,59,60]. Following injury, acute inflammation immediately ensues by triggering MC degranulation of pro-inflammatory mediators, such as histamine and VEGF, which increases vascular permeability. Studies in MC-deficient mice have demonstrated that this increase in vascular permeability is MC-dependent [56]. Importantly, during the inflammatory phase immune cells secrete other pro-angiogenic mediators to induce vascularization. For example, other than VEGF, MCs also release platelet derived growth factor (PDGF) and fibroblast growth factor 2 (FGF-2) to promote angiogenesis [61,62,63,64,65]. In addition, the preformed serine proteases (*i.e.*, tryptase) stored in the secretory granules of MCs stimulates vascular tube formation and proliferation of endothelial cells *in vitro* [50,66]. Finally, the pre-synthesized matrix metalloproteinase (MMPs) stored within their granules enables MCs to indirectly modulate angiogenesis by releasing MMPs (e.g., MMP-2 and MMP-9) to cleave and release matrix-bound angiogenic factors to the injured site.

Interestingly, it has long been recognized that MCs may play a role in fracture healing [67]. Histology studies of fractures in a rat model revealed that in the first two weeks, MCs are found either in the vicinity of blood vessels or in the vascularized tissue proliferating into the cartilaginous portion of subperiosteal callus [68]. This finding led to the view that MCs are involved in digestion of extracellular matrix and angiogenesis in the early stages of fracture healing, which remains an untested hypothesis.

### 3.3. The Role of Mast Cells in Tissue Fibrosis

Fibrosis is the formation of excessive fibrous connective tissue, namely collagen and glycoaminoglycans (GAGs), in response to injury or disease. The excess connective tissue, deposited by activated tissue fibroblasts, results in the impairment of the physiological function of the tissue [69]. The role of MCs in fibrosis has been long known to play a pivotal part in various organs, such as the heart, kidney, liver, lungs, and various autoimmune pathologies, such as systemic sclerosis [62,70,71,72,73,74]. Indeed, MCs haven been shown to promote fibrosis through both direct and indirect, fibroblast-stimulating mechanisms. Mechanisms implicating MCs in fibrosis include degranulation of preformed profibrotic mediators including transforming growth factor beta (TGF-β), PDGF, histamine, chymase, tryptase, and GAGs [52,75]. The secretion of TGF-β drives collagen production by activated fibroblasts, which works in concert with the release of GAGs by MCs to promote scar formation through remodeling of the extracellular matrix milieu in the interstitial tissue. Furthermore, histamine and tryptase secretion promotes the proliferation and differentiation of fibroblasts into contractile myofibroblasts [76,77,78,79,80] while the release of chymase (the primary enzymatic component of MCs) stimulates the production of collagen fibrils through cleavage of procollagen type I [81,82]. Importantly, another recently proposed mechanism of fibrosis includes the direct interactions of fibroblasts and MCs via gap junctions. Although not much is known about these intercellular interactions, it has been shown to induce fibroblast proliferation and myofibroblast differentiation and contraction postulated to operate via reciprocal cell activation [83,84,85,86].

### 3.4. The Role of Large Vessel-Associated Mast Cells in Critical Defect Healing

**Figure 1:**
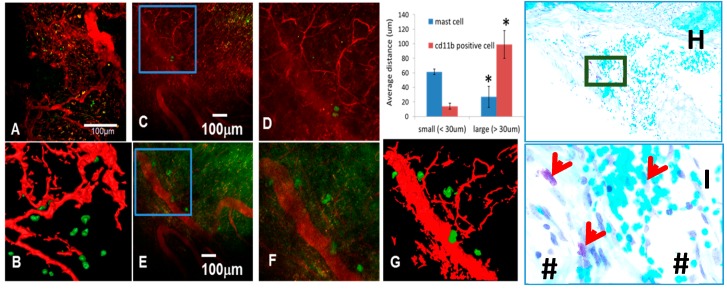
Mast cells accumulate proximal to large vessels in fibrotic tissue of healing critical defects. The murine chronic cranial defect window model was utilized for *in vivo* multiphoton laser scanning microscopy (MPLSM) to assess the temporal-spatial relationship of arteriogenesis and mast cell accumulation during critical bone defect healing. MPLSM was performed on mice (*n* = 5) with cranial defect windows following administration of i.v. Texas red dextran (TRD) and APC-conjugated anti-CD11b or FITC conjugated anti-Mcpt5 antibodies. (**A**) Representative 10× field of the CD11b+ cells (**green**) and vasculature (**red**) with a 3D reconstruction (**B**), are shown to illustrate that monocytes and macrophages primarily exist proximal to small vessels within the critical defect after three weeks of healing; In contrast, few Mcpt5+ mast cells are found near small vessels ((**C**); boxed region is (**D**)); but appear in immediate proximity to large vessels ((**E**); boxed region is (**F**) and 3D reconstructed image is (**G**)); MATLAB quantification of the distance of the labeled cells from small and large vessels is presented as mean +/− SD (* *p* < 0.05 *vs.* small vessels). Histologic confirmation of these findings is provided by toluidine blue staining of the calvaria tissue presented at 5× (**H**); and boxed region at 20× (**I**); in which the granulated mast cells (**red** arrows) stain purple in immediate proximity to large blood vessels (#).

In addition to the finding that rPTH therapy inhibits arteriogenesis during allograft healing, it was observed that MCs were absent from the soft tissues around healing allografts, which were prominently present in placebo-treated mice [42]. This is believed to be an indirect effect of rPTH, since PTH receptor expression has not been detected on mast cells. Moreover, toluidine blue-stained histology of the placebo allografts revealed that these MCs were primarily located adjacent to large blood vessels. To confirm this observation, which implicates large vessel-associated MCs in the fibrotic cascade of non-healing allografts, a murine chronic cranial defect window model with *in vivo* multiphoton laser scanning microscopy (MPLSM) was recently employed [87] to assess the temporal-spatial relationship of arteriogenesis and MC accumulation during critical bone defect healing. To perform this intravital fluorescent microscopy, labeled antibodies against the MC-specific surface marker mast cell protease (Mcpt) 5 [88], and a label control antibody against CD11b that recognizes monocytes and macrophages, were used. The results from these preliminary studies demonstrate that in contrast to random distribution of CD11b+ cells in a healing critical defect, MCs accumulate in immediate proximity to large blood vessels (Figure 1). One major question that is currently under investigation is the mechanism for reduction of MC accumulation during rPTH therapy. We speculate that vascular smooth muscle cells produce MC differentiation and/or survival factors (*i.e.*, stem cell factor) and, thus, rPTH inhibition of arteriogenesis results in decreased MC due to the absence of critical factors from smooth muscle cells.

**Figure 2:**
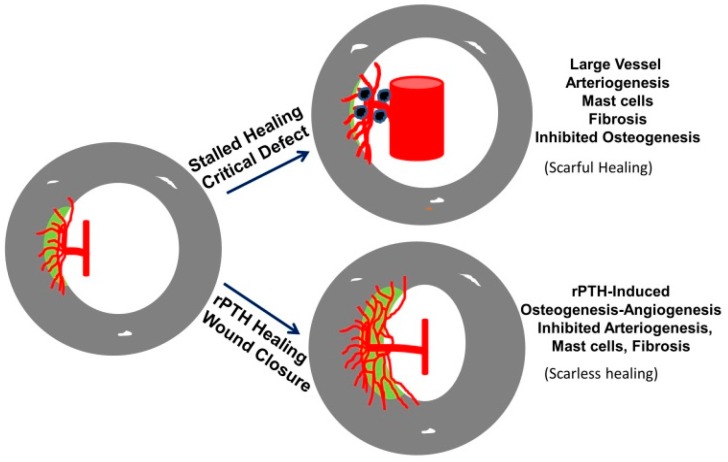
Schematic Model of Scarful *vs.* Scarless Healing and the Adjuvant Effects of rPTH Therapy. An emerging model to explain the fundamental basis of a non-healing critical defect in bone posits that there is a temporal-spatial competition between coupled small vessel angiogenesis-osteogenesis at the leading edge of the healing defect, and large vessel arteriogenesis-fibrosis within the defect. In the first two weeks after injury small vessel (<10 mm) angiogenesis facilitates rapid defect filling (scarless healing) from the leading edge (~0.02 mm/day) via proliferating/migrating bone forming osteoblasts (green). Simultaneously, large vessel (>30 mm) arteriogenesis occurs with the appearance of pro-fibrotic mast cells (dark blue) in the center of the defect, which completely inhibits osteoblastic defect filling (0 mm/day) thereafter, resulting in “scarful healing” of the critical defect. Moreover, rPTH therapy facilitates critical defect healing by: (1) its well-known anabolic effects on osteogenic cells to increase defect filling at the healing front; (2) coupled osteogenic cell-induced small vessel angiogenesis at the healing front; and (3) inhibition of large vessel arteriogenesis/fibrosis within the defect, resulting in scarless healing and ultimate wound closure.

Taken together, these observations suggest that there are pivotal roles for arteriogenesis and MCs in the fibrotic cascade of critical defect and massive allograft “scarful” healing, which does not occur in “scarless” autograft or rPTH treated allograft healing (Figure 2). In this model, the first two weeks of healing are characterized by small vessel (<10 μm) angiogenesis, which facilitates rapid defect filling (scarless healing) from the leading edge (~0.02 mm/day) via proliferating/migrating bone forming osteoblasts. Simultaneously, Ang-2 dependent large vessel (>30 μm) arteriogenesis occurs with the appearance of pro-fibrotic MCs in the hypoxic center of the critical defect, which completely inhibits osteoblastic defect filling (0 mm/day) thereafter, resulting in “scarful healing” of the critical defect. It is also postulated that when indicated, rPTH therapy is an adjuvant bioengineering solution for critical defect healing by: (1) its well-known anabolic effects on osteogenic cells to increase defect filling at the healing front; (2) coupled osteogenic cell-induced small vessel angiogenesis at the healing front; and (3) inhibition of large vessel arteriogenesis/fibrosis within the defect, with a decrease in total vascular volume, resulting in Scarless healing and ultimate wound closure.

## 4. Conclusions

As bioengineering solutions are needed to address the significant unmet clinical needs of critical size defects, here we propose that arteriogenesis and mast cells are underappreciated drug targets for adjuvant therapy. We also propose teriparatide is an effective adjuvant therapy, largely due to its inhibitory effects on arteriogenesis and MCs (see summary in Table 1). Unfortunately, theoretical safety concerns have led to FDA contraindications for teriparatide therapy in cancer patients and children, which are two significant patient populations that undergo critical bone defects due to craniomaxillofacial reconstruction and limb salvage surgery. However, there is hope that this “black box warning” may be removed based on the absence of substantiated clinical findings including: (1) the FDA mandated 15-year post-marketing osteosarcoma surveillance study, which has not detected a pattern indicative of a causal association between teriparatide treatment and osteosarcoma in humans at the halfway point [89]; (2) phase 2 clinical trials that have demonstrated rPTH safety and efficacy on adult fractures [90,91,92]; and (3) rPTH therapy safety and efficacy has been demonstrated in patients with alveolar bone defects and osseous wound healing in the oral cavity [93]. Alternatively, other than teriparatide, adjuvant therapy could be accomplished via direct inhibition of arteriogenesis with Ang-2 inhibitors (e.g., AMG-386), and MCs with currently available agents (e.g., cromolyn sodium). Yet, future studies are required to establish the safety and efficacy of this promising approach.

**Table 1:** A summary of bioengineering solutions for critical bone defect repair and regeneration.

Approach	Mechanism	Advantages	Disadvantages
Autografts	Osteoinduction; osteoconduction; and osteogenesis	Histoidentical; stimulates a robust regenerative response (high union ratios)	Finite amount; co-site morbidities; post-operative pain; and infection
Allografts	Osteoconduction	High availability and accessibility; circumvent donor site morbidity; reduced surgical time and site	Disease transmission; high failure rates; infection; immunologic reaction; and low union ratios
Coated Allografts	Release of inductive agents on an osteoconductive matrix	All advantages of allografts plus choice of coated agents; stimulates a robust regenerative response (high union ratios)	All disadvantages of allografts plus dependency on release of coated agents; laborious preparation; not FDA-approved
Intermittent rPTH	Anabolic effects on osteogenic cells; promotes small-vessel angiogenesis; inhibits arteriogenesis and mast cell accretion (early evidence)	FDA-approved adjuvant therapy; inhibits scar formation; stimulates a robust regenerative response (high union ratios)	Contra-indicated in a large target population (children and cancer patients)

## References

[1] Reizner W., Hunter J.G., O’Malley N.T., Southgate R.D., Schwarz E.M., Kates S.L. (2014). A systematic review of animal models for staphylococcus aureus osteomyelitis. Eur. Cell Mater..

[2] Harris J.S., Bemenderfer T.B., Wessel A.R., Kacena M.A. (2013). A review of mouse critical size defect models in weight bearing bones. Bone.

[3] Marsell R., Einhorn T.A. (2011). The biology of fracture healing. Injury.

[4] Carano R.A., Filvaroff E.H. (2003). Angiogenesis and bone repair. Drug Discov. Today.

[5] Kanczler J.M., Oreffo R.O. (2008). Osteogenesis and angiogenesis: The potential for engineering bone. Eur. Cell Mater..

[6] Giannotti S., Bottai V., Dell’osso G., Pini E., de Paola G., Bugelli G., Guido G. (2013). Current medical treatment strategies concerning fracture healing. Clin. Cases Miner. Bone Metab..

[7] Schipani E., Maes C., Carmeliet G., Semenza G.L. (2009). Regulation of osteogenesis-angiogenesis coupling by HIFs and VEGF. J. Bone Miner. Res..

[8] Bosse M.J., MacKenzie E.J., Kellam J.F., Burgess A.R., Webb L.X., Swiontkowski M.F., Sanders R.W., Jones A.L., McAndrew M.P., Patterson B.M. (2002). An analysis of outcomes of reconstruction or amputation after leg-threatening injuries. N. Engl. J. Med..

[9] Marino J.T., Ziran B.H. (2010). Use of solid and cancellous autologous bone graft for fractures and nonunions. Orthop. Clin. N. Am..

[10] Wheeler D.L., Enneking W.F. (2005). Allograft bone decreases in strength in vivo over time. Clin. Orthop. Relat. Res..

[11] Kleinhans C., Mohan R.R., Vacun G., Schwarz T., Haller B., Sun Y., Kahlig A., Kluger P., Finne-Wistrand A., Walles H. (2015). A perfusion bioreactor system efficiently generates cell-loaded bone substitute materials for addressing critical size bone defects. Biotechnol. J..

[12] Herrmann M., Verrier S., Alini M. (2015). Strategies to stimulate mobilization and homing of endogenous stem and progenitor cells for bone tissue repair. Front. Bioeng. Biotechnol..

[13] Gothard D., Smith E.L., Kanczler J.M., Rashidi H., Qutachi O., Henstock J., Rotherham M., el Haj A., Shakesheff K.M., Oreffo R.O. (2014). Tissue engineered bone using select growth factors: A comprehensive review of animal studies and clinical translation studies in man. Eur. Cell Mater..

[14] Amini A.R., Laurencin C.T., Nukavarapu S.P. (2012). Bone tissue engineering: Recent advances and challenges. Crit. Rev. Biomed. Eng..

[15] Das A., Segar C.E., Chu Y., Wang T.W., Lin Y., Yang C., Du X., Ogle R.C., Cui Q., Botchwey E.A. (2015). Bioactive lipid coating of bone allografts directs engraftment and fate determination of bone marrow-derived cells in rat gfp chimeras. Biomaterials.

[16] Wang T., Krieger J., Huang C., Das A., Francis M.P., Ogle R., Botchwey E. (2015). Enhanced osseous integration of human trabecular allografts following surface modification with bioactive lipids. Drug Deliv. Transl. Res..

[17] Ito H., Koefoed M., Tiyapatanaputi P., Gromov K., Goater J.J., Carmouche J., Zhang X., Rubery P.T., Rabinowitz J., Samulski R.J. (2005). Remodeling of cortical bone allografts mediated by adherent rAAV-RANKL and VEGF gene therapy. Nat. Med..

[18] Yazici C., Takahata M., Reynolds D.G., Xie C., Samulski R.J., Samulski J., Beecham E.J., Gertzman A.A., Spilker M., Zhang X. (2011). Self-complementary AAV2.5-BMP2-coated femoral allografts mediated superior bone healing versus live autografts in mice with equivalent biomechanics to unfractured femur. Mol. Ther..

[19] Pelled G., Ben-Arav A., Hock C., Reynolds D.G., Yazici C., Zilberman Y., Gazit Z., Awad H., Gazit D., Schwarz E.M. (2010). Direct gene therapy for bone regeneration: Gene delivery, animal models, and outcome measures. Tissue Eng. Part. B Rev..

[20] Ben-Arav A., Pelled G., Zilberman Y., Kimelman-Bleich N., Gazit Z., Schwarz E.M., Gazit D. (2012). Adeno-associated virus-coated allografts: A novel approach for cranioplasty. J. Tissue Eng. Regen. Med..

[21] Yasuda H., Yano K., Wakitani S., Matsumoto T., Nakamura H., Takaoka K. (2012). Repair of critical long bone defects using frozen bone allografts coated with an rhBMP-2-retaining paste. J. Orthop. Sci..

[22] Jones C.B., Sabatino C.T., Badura J.M., Sietsema D.L., Marotta J.S. (2008). Improved healing efficacy in canine ulnar segmental defects with increasing recombinant human bone morphogenetic protein-2/allograft ratios. J. Orthop. Trauma.

[23] Baas J., Elmengaard B., Jensen T.B., Jakobsen T., Andersen N.T., Soballe K. (2008). The effect of pretreating morselized allograft bone with rhBMP-2 and/or pamidronate on the fixation of porous Ti and HA-coated implants. Biomaterials.

[24] Sharmin F., Adams D., Pensak M., Dukas A., Lieberman J., Khan Y. (2015). Biofunctionalizing devitalized bone allografts through polymer-mediated short and long term growth factor delivery. J. Biomed. Mater. Res. A.

[25] Zhang X., Xie C., Lin A.S., Ito H., Awad H., Lieberman J.R., Rubery P.T., Schwarz E.M., O’Keefe R.J., Guldberg R.E. (2005). Periosteal progenitor cell fate in segmental cortical bone graft transplantations: Implications for functional tissue engineering. J. Bone Miner. Res..

[26] Street J., Bao M., deGuzman L., Bunting S., Peale F.V., Ferrara N., Steinmetz H., Hoeffel J., Cleland J.L., Daugherty A. (2002). Vascular endothelial growth factor stimulates bone repair by promoting angiogenesis and bone turnover. Proc. Natl. Acad. Sci. USA.

[27] Kronenberg H.M. (2003). Developmental regulation of the growth plate. Nature.

[28] Berg C., Neumeyer K., Kirkpatrick P. (2003). Teriparatide. Nat. Rev. Drug Discov..

[29] Jiang Y., Zhao J.J., Mitlak B.H., Wang O., Genant H.K., Eriksen E.F. (2003). Recombinant human parathyroid hormone (1–34) [Teriparatide] improves both cortical and cancellous bone structure. J. Bone Miner. Res..

[30] Lindsay R., Zhou H., Cosman F., Nieves J., Dempster D.W., Hodsman A.B. (2007). Effects of a one-month treatment with PTH(1–34) on bone formation on cancellous, endocortical, and periosteal surfaces of the human ilium. J. Bone Miner. Res..

[31] Rehman Q., Lang T.F., Arnaud C.D., Modin G.W., Lane N.E. (2003). Daily treatment with parathyroid hormone is associated with an increase in vertebral cross-sectional area in postmenopausal women with glucocorticoid-induced osteoporosis. Osteoporos. Int..

[32] Hodsman A.B., Bauer D.C., Dempster D.W., Dian L., Hanley D.A., Harris S.T., Kendler D.L., McClung M.R., Miller P.D., Olszynski W.P. (2005). Parathyroid hormone and teriparatide for the treatment of osteoporosis: A review of the evidence and suggested guidelines for its use. Endocr. Rev..

[33] Girotra M., Rubin M.R., Bilezikian J.P. (2006). The use of parathyroid hormone in the treatment of osteoporosis. Rev. Endocr. Metab. Disord..

[34] Boonen S., Marin F., Mellstrom D., Xie L., Desaiah D., Krege J.H., Rosen C.J. (2006). Safety and efficacy of teriparatide in elderly women with established osteoporosis: Bone anabolic therapy from a geriatric perspective. J. Am. Geriatr. Soc..

[35] Kaback L.A., Soung-Do Y., Naik A., Geneau G., Schwarz E.M., Rosier R.N., O’Keefe R.J., Drissi H. (2008). Teriparatide (1–34 human PTH) regulation of osterix during fracture repair. J. Cell Biochem..

[36] Yu B., Zhao X., Yang C., Crane J., Xian L., Lu W., Wan M., Cao X. (2012). Parathyroid hormone induces differentiation of mesenchymal stromal/stem cells by enhancing bone morphogenetic protein signaling. J. Bone Miner. Res..

[37] Carpio L., Gladu J., Goltzman D., Rabbani S.A. (2001). Induction of osteoblast differentiation indexes by PTHrP in MG-63 cells involves multiple signaling pathways. Am. J. Physiol. Endocrinol. Metab..

[38] Daugaard H., Elmengaard B., Andreassen T.T., Baas J., Bechtold J.E., Soballe K. (2011). The combined effect of parathyroid hormone and bone graft on implant fixation. J. Bone Jt. Surg. Br..

[39] Reynolds D.G., Takahata M., Lerner A.L., O’Keefe R.J., Schwarz E.M., Awad H.A. (2011). Teriparatide therapy enhances devitalized femoral allograft osseointegration and biomechanics in a murine model. Bone.

[40] Yamamoto Y., Washimi Y., Kanaji A., Tajima K., Ishimura D., Yamada H. (2012). The effect of bisphosphonate and intermittent human parathyroid hormone 1–34 treatments on cortical bone allografts in rabbits. J. Endocrinol. Investig..

[41] Takahata M., Schwarz E.M., Chen T., O’Keefe R.J., Awad H.A. (2012). Delayed short course treatment with teriparatide (PTH(1–34) ) improves femoral allograft healing by enhancing intramembranous bone formation at the graft-host junction. J. Bone Miner. Res..

[42] Dhillon R.S., Xie C., Tyler W., Calvi L.M., Awad H.A., Zuscik M.J., O’Keefe R.J., Schwarz E.M. (2013). PTH enhanced structural allograft healing is associated with decreased angiopoietin-2 mediated arteriogenesis, mast cell accumulation and fibrosis. J. Bone Miner. Res..

[43] Abzhanov A., Rodda S.J., McMahon A.P., Tabin C.J. (2007). Regulation of skeletogenic differentiation in cranial dermal bone. Development.

[44] Sheyn D., Cohn-Yakubovich D., Kallai I., Su S., Da X., Pelled G., Tawackoli W., Cook-Weins G., Schwarz E.M., Gazit D. (2013). Pth promotes allograft integration in a calvarial bone defect. Mol. Pharm..

[45] Barnes G.L., Kakar S., Vora S., Morgan E.F., Gerstenfeld L.C., Einhorn T.A. (2008). Stimulation of fracture-healing with systemic intermittent parathyroid hormone treatment. J. Bone Jt. Surg. Am..

[46] Reynolds D.G., Shaikh S., Papuga M.O., Lerner A.L., O’Keefe R.J., Schwarz E.M., Awad H.A. (2009). MuCT-based measurement of cortical bone graft-to-host union. J. Bone Miner. Res..

[47] Rubery P.T., Bukata S.V. (2010). Teriparatide may accelerate healing in delayed unions of type III odontoid fractures: A report of 3 cases. J. Spinal. Disord. Tech..

[48] Kang S.Y., Deshpande S.S., Donneys A., Rodriguez J.J., Nelson N.S., Felice P.A., Chepeha D.B., Buchman S.R. (2013). Parathyroid hormone reverses radiation induced hypovascularity in a murine model of distraction osteogenesis. Bone.

[49] Ribatti D., Crivellato E. (2009). The controversial role of mast cells in tumor growth. Int. Rev. Cell Mol. Biol..

[50] Metcalfe D.D., Baram D., Mekori Y.A. (1997). Mast cells. Physiol. Rev..

[51] Rodewald H.R., Feyerabend T.B. (2012). Widespread immunological functions of mast cells: Fact or fiction?. Immunity.

[52] Wulff B.C., Wilgus T.A. (2013). Mast cell activity in the healing wound: More than meets the eye?. Exp. Dermatol.

[53] Oschatz C., Maas C., Lecher B., Jansen T., Bjorkqvist J., Tradler T., Sedlmeier R., Burfeind P., Cichon S., Hammerschmidt S. (2011). Mast cells increase vascular permeability by heparin-initiated bradykinin formation *in vivo*. Immunity.

[54] Kunder C.A., St John A.L., Abraham S.N. (2011). Mast cell modulation of the vascular and lymphatic endothelium. Blood.

[55] Gilfillan A.M., Beaven M.A. (2011). Regulation of mast cell responses in health and disease. Crit. Rev. Immunol..

[56] Weller K., Foitzik K., Paus R., Syska W., Maurer M. (2006). Mast cells are required for normal healing of skin wounds in mice. FASEB J..

[57] Egozi E.I., Ferreira A.M., Burns A.L., Gamelli R.L., Dipietro L.A. (2003). Mast cells modulate the inflammatory but not the proliferative response in healing wounds. Wound Repair Regen..

[58] Lin L., Bankaitis E., Heimbach L., Li N., Abrink M., Pejler G., An L., Diaz L.A., Werb Z., Liu Z. (2011). Dual targets for mouse mast cell protease-4 in mediating tissue damage in experimental bullous pemphigoid. J. Biol. Chem..

[59] Takato H., Yasui M., Ichikawa Y., Waseda Y., Inuzuka K., Nishizawa Y., Tagami A., Fujimura M., Nakao S. (2011). The specific chymase inhibitor TY-51469 suppresses the accumulation of neutrophils in the lung and reduces silica-induced pulmonary fibrosis in mice. Exp. Lung Res..

[60] Younan G., Suber F., Xing W., Shi T., Kunori Y., Abrink M., Pejler G., Schlenner S.M., Rodewald H.R., Moore F.D. (2010). The inflammatory response after an epidermal burn depends on the activities of mouse mast cell proteases 4 and 5. J. Immunol..

[61] Shiota N., Nishikori Y., Kakizoe E., Shimoura K., Niibayashi T., Shimbori C., Tanaka T., Okunishi H. (2010). Pathophysiological role of skin mast cells in wound healing after scald injury: Study with mast cell-deficient W/W(V) mice. Int. Arch. Allergy Immunol..

[62] Qu Z., Liebler J.M., Powers M.R., Galey T., Ahmadi P., Huang X.N., Ansel J.C., Butterfield J.H., Planck S.R., Rosenbaum J.T. (1995). Mast cells are a major source of basic fibroblast growth factor in chronic inflammation and cutaneous hemangioma. Am. J. Pathol.

[63] Qu Z., Huang X., Ahmadi P., Stenberg P., Liebler J.M., Le A.C., Planck S.R., Rosenbaum J.T. (1998). Synthesis of basic fibroblast growth factor by murine mast cells. Regulation by transforming growth factor beta, tumor necrosis factor alpha, and stem cell factor. Int. Arch. Allergy Immunol..

[64] Qu Z., Kayton R.J., Ahmadi P., Liebler J.M., Powers M.R., Planck S.R., Rosenbaum J.T. (1998). Ultrastructural immunolocalization of basic fibroblast growth factor in mast cell secretory granules. Morphological evidence for bFGF release through degranulation. J. Histochem. Cytochem..

[65] Grutzkau A., Kruger-Krasagakes S., Baumeister H., Schwarz C., Kogel H., Welker P., Lippert U., Henz B.M., Moller A. (1998). Synthesis, storage, and release of vascular endothelial growth factor/vascular permeability factor (VEGF/VPF) by human mast cells: Implications for the biological significance of VEGF206. Mol. Biol. Cell..

[66] Blair R.J., Meng H., Marchese M.J., Ren S., Schwartz L.B., Tonnesen M.G., Gruber B.L. (1997). Human mast cells stimulate vascular tube formation. Tryptase is a novel, potent angiogenic factor. J. Clin. Invest..

[67] Lindholm R., Lindholm S., Liukko P. (1967). Fracture healing and mast cells. I. The periosteal callus in rats. Acta Orthop. Scand..

[68] Banovac K., Renfree K., Makowski A.L., Latta L.L., Altman R.D. (1995). Fracture healing and mast cells. J. Orthop. Trauma.

[69] Gruber B.L. (2003). Mast cells in the pathogenesis of fibrosis. Curr. Rheumatol. Rep..

[70] Abe M., Yokoyama Y., Amano H., Matsushima Y., Kan C., Ishikawa O. (2002). Effect of activated human mast cells and mast cell-derived mediators on proliferation, type I collagen production and glycosaminoglycans synthesis by human dermal fibroblasts. Eur. J. Dermatol..

[71] Hirai S., Ohyane C., Kim Y.I., Lin S., Goto T., Takahashi N., Kim C.S., Kang J., Yu R., Kawada T. (2014). Involvement of mast cells in adipose tissue fibrosis. Am. J. Physiol. Endocrinol. Metab..

[72] Summers S.A., Gan P.Y., Dewage L., Ma F.T., Ooi J.D., O’Sullivan K.M., Nikolic-Paterson D.J., Kitching A.R., Holdsworth S.R. (2012). Mast cell activation and degranulation promotes renal fibrosis in experimental unilateral ureteric obstruction. Kidney Int..

[73] Erjefalt J.S. (2014). Mast cells in human airways: The culprit?. Eur. Respir. Rev..

[74] Ningyan G., Xu Y., Hongfei S., Jingjing C., Min C. (2015). The role of macrophage migration inhibitory factor in mast cell-stimulated fibroblast proliferation and collagen production. PLoS ONE.

[75] Hugle T. (2014). Beyond allergy: The role of mast cells in fibrosis. Swiss Med. Wkly..

[76] Gailit J., Marchese M.J., Kew R.R., Gruber B.L. (2001). The differentiation and function of myofibroblasts is regulated by mast cell mediators. J. Invest. Dermatol..

[77] Hatamochi A., Fujiwara K., Ueki H. (1985). Effects of histamine on collagen synthesis by cultured fibroblasts derived from guinea pig skin. Arch. Dermatol. Res..

[78] Kupietzky A., Levi-Schaffer F. (1996). The role of mast cell-derived histamine in the closure of an *in vitro* wound. Inflamm. Res..

[79] Gruber B.L., Kew R.R., Jelaska A., Marchese M.J., Garlick J., Ren S., Schwartz L.B., Korn J.H. (1997). Human mast cells activate fibroblasts: Tryptase is a fibrogenic factor stimulating collagen messenger ribonucleic acid synthesis and fibroblast chemotaxis. J. Immunol..

[80] Albrecht M., Frungieri M.B., Kunz L., Ramsch R., Meineke V., Kohn F.M., Mayerhofer A. (2005). Divergent effects of the major mast cell products histamine, tryptase and TNF-alpha on human fibroblast behaviour. Cell. Mol. Life Sci..

[81] Hermes B., Feldmann-Boddeker I., Welker P., Algermissen B., Steckelings M.U., Grabbe J., Henz B.M. (2000). Altered expression of mast cell chymase and tryptase and of c-Kit in human cutaneous scar tissue. J. Investig. Dermatol..

[82] Kofford M.W., Schwartz L.B., Schechter N.M., Yager D.R., Diegelmann R.F., Graham M.F. (1997). Cleavage of type I procollagen by human mast cell chymase initiates collagen fibril formation and generates a unique carboxyl-terminal propeptide. J. Biol. Chem..

[83] Pistorio A.L., Ehrlich H.P. (2011). Modulatory effects of connexin-43 expression on gap junction intercellular communications with mast cells and fibroblasts. J. Cell. Biochem..

[84] Au S.R., Au K., Saggers G.C., Karne N., Ehrlich H.P. (2007). Rat mast cells communicate with fibroblasts via gap junction intercellular communications. J. Cell. Biochem..

[85] Moyer K.E., Saggers G.C., Ehrlich H.P. (2004). Mast cells promote fibroblast populated collagen lattice contraction through gap junction intercellular communication. Wound Repair Regen..

[86] Foley T.T., Saggers G.C., Moyer K.E., Ehrlich H.P. (2011). Rat mast cells enhance fibroblast proliferation and fibroblast-populated collagen lattice contraction through gap junctional intercellular communications. Plast. Reconstr. Surg..

[87] Huang C., Ness V.P., Yang X., Chen H., Luo J., Brown E.B., Zhang X. (2015). Spatiotemporal analyses of osteogenesis and angiogenesis via intravital imaging in cranial bone defect repair. J. Bone Miner. Res..

[88] Scholten J., Hartmann K., Gerbaulet A., Krieg T., Muller W., Testa G., Roers A. (2008). Mast cell-specific Cre/loxP-mediated recombination *in vivo*. Transgenic Res..

[89] Andrews E.B., Gilsenan A.W., Midkiff K., Sherrill B., Wu Y., Mann B.H., Masica D. (2012). The us postmarketing surveillance study of adult osteosarcoma and teriparatide: Study design and findings from the first 7 years. J. Bone Miner. Res..

[90] Aspenberg P., Genant H.K., Johansson T., Nino A.J., See K., Krohn K., Garcia-Hernandez P.A., Recknor C.P., Einhorn T.A., Dalsky G.P. (2009). Teriparatide for acceleration of fracture repair in humans: A prospective, randomized, double-blind study of 102 postmenopausal women with distal radial fractures. J. Bone Miner. Res..

[91] Aspenberg P., Johansson T. (2010). Teriparatide improves early callus formation in distal radial fractures. Acta Orthop..

[92] Peichl P., Holzer L.A., Maier R., Holzer G. (2011). Parathyroid hormone 1–84 accelerates fracture-healing in pubic bones of elderly osteoporotic women. J. Bone Jt. Surg. Am..

[93] Bashutski J.D., Eber R.M., Kinney J.S., Benavides E., Maitra S., Braun T.M., Giannobile W.V., McCauley L.K. (2010). Teriparatide and osseous regeneration in the oral cavity. N. Engl. J. Med..

